# Time abides long enough for those who make use of it

**DOI:** 10.1186/s41199-018-0038-4

**Published:** 2018-12-19

**Authors:** Muhammad M. Fareed, Thomas J. Galloway

**Affiliations:** Department of Radiation Oncology, Fox Chase Cancer Center, 333 Cottman Avenue, Philadelphia, PA 19111 USA

## Abstract

Increased treatment package time is an independent poor prognostic factor for outcomes in head and neck squamous cell carcinoma (HNSCC). Similarly the timeliness of treatment initiation is a risk factor for disease recurrence. Despite these well-known issues, the timeliness of treatment initiation is actually worsening in the United States and the expeditious completion of radiation treatments continues to be difficult secondary to a number of patients and treatment related issues. This analysis evaluates the current data on treatment intervals in the management of head and neck cancer. Rapid staging/diagnosis of head and neck cancer, appropriate referrals to providers qualified to treat said cancer, and expeditious treatment completion remains the most cost-effective, widely applicable method to improve outcomes in head and neck cancer.

Time abides long enough for those who make use of it – Leonardo Da Vinci

## Introduction

Increased treatment package time (defined as duration between the initiation and completion of curative therapy), increased time to treatment initiation (TTI – defined as the duration between the histologic diagnosis and initiation of treatment) and increased radiation treatment time (RTT – defined as the duration between the first and last fraction of external beam radiation) have all been demonstrated to be detrimental in the management of head and neck squamous cell carcinoma (HNSCC). In addition, prolonged TTI affects patient satisfaction and quality of life [1] and exacerbates the psychosocial distress accompanying a cancer diagnosis [2, 3]. Furthermore, while most prolongations of both package time and RTT are unplanned, they may be increasing due to increasingly complex treatment regimens [4] that are more toxic than prior regimens [5]. Thus, although the effects of timing in head and neck cancer have been discussed for decades [6–8], a critical reappraisal of the lessons of timing is indicated. In this manuscript we will review the timing of each interval associated with the successful treatment of head and neck cancer and study the impacts on treatment outcomes. The time between first appreciation of symptom(s) related to an underlying cancer and initial presentation to an oncology provider is an extremely difficult interval to quantify and examine and will not be evaluated in this monograph.

### Time to treatment initiation

Many different types of evidence suggest that it is prudent to avoid the prolongation of initiating potentially curative radiation therapy [9] and cancer surgery [10]. There is overwhelming evidence that most human cancers steadily grow and progress and although the growth rate is variable [11, 12] this is a provocative rationale to expedite treatment starts. Increase prolongation of TTI leads to stage progression and larger tumors and an associated decrease in recurrence free survival [13]. However convincing these common sense arguments may seem, successfully limiting the interval between diagnosis and treatment start and quantifying the effects of prolongation of that time frame is difficult.

Historically, increased TTI was a consequence of an imbalance of supply and demand. Largely due to an increase in the use of radiation resources for breast and prostate cancers [14], radiotherapy centers on different continents reported the same problem some decades ago – a prolongation of the time between diagnosis and treatment start [15, 16]. In the United States the response to this was more access – more radiation oncologists and more radiation therapy centers [17]. However, recent series suggest that even with expanding access to radiation oncologists TTI is not only still elevated, but actively increasing [4]. Understanding why TTI is still elevated and barriers to its reduction requires an understanding of the processes involved in expeditious yet accurate treatment initiation.

Reported TTI evaluations of patients treated with curative radiotherapy demonstrate a remarkably similar trend (Table 1) – patients generally start radiotherapy a month after the histologic diagnosis is established by a biopsy. This is remarkable – while some impediments to treatment start are constant (it takes time to interpret the biopsy, arrange a new patient referral with a radiation oncologist, schedule appointments), the management of head and neck tumors with primary radiation is profoundly different in 2013 than it was in 1965. As referenced earlier, prolonged TTI was once referable to capacity. While factors related to access unfortunately continue to predict for increasing TTI in the United States [4], the increasing complexity of care has taken over as an additional driver of increased TTI. In 2001 Medicare began to cover ^18F^fluorodeoxyglucose/positron emission tomography (FDG/PET) when used for the diagnosis, staging, and restaging of head and neck cancers [18]. Subsequent prospective investigations demonstrated that pre-therapy PET/computed tomography (CT) imaging could alter the planned management of head and neck cancer [19]. Clinicians responded by ordering more PET/CT scans for the management of head and neck cancer [20]. The increased use of intensity modulated radiation therapy (IMRT) from < 10% in 1998 [21] to near ubiquity in 2018 requires days of additional time for contouring, planning, and quality assurance that prolongs TTI [22]. In addition to these clinical factors additional delays stem from the increasing need for time-consuming prior authorizations for medications, additional tests, and referrals [23]. Data from the United States suggests that the increasing complexity of care should not be ignored – the median TTI for all patients increased from 19 days to 30 days from 1998 and 2011 [4].

**Table 1:** Time to treatment initiation (TTI) Evaluations

	Data Source	Years	n	Site	Median TTI	HR^a^
Fortin A [25]	Retrospective single institution	1988–1997	623	T1-2 N0 HNC	1989: 28 d	1.45 (1.06–1.97)
				55% Larynx	1995: 46 d	Delay > 30 d
					1997: 55 d	
Leon X [23]	Retrospective single institution	1985–1998	797	All HNC	44 d	0.99 (0.83–1.19)
				45% Larynx		Delay > 33 d
Hansen O [13]	Retrospective single institution	1965–1997	611	Stage I-III	21 d	1.16 (0.84–1.59)
				Glottic Cancer		Continuous
Caudell J [24]	Retrospective single institution	1995–2007	427	Stage III-IV HNC	34 d	1.002 (0.999–1.005)
				55% Oropharynx		Continuous
Liang H [22]	Retrospective single institution	1998–2013	9896	Stage I-IV NPC	RT: 20 d	1.13 (1.04–1.23)
				45% Stage III	CRT: 22 d	Delay > 30 d
Murphy CT [26]	Prospective Database	2003–2006	51,655	All HNC	26 d	1.08 (1.03–1.13)
				42% Larynx		Delay 61–90 d

*HNC* = head and neck cancer, *RT* = RT alone, *CRT* = chemoradiation, *d* = days

^a^HR is a comparison of survival as a function of increased TTI. Comparison is TTI ≤ 30 days v the interval specified or an evaluation of increased TTI as a continuous variable

Quantifying TTI is straightforward. Patients have a date of biopsy and a date of treatment start – the difference is the TTI. Evaluating the impact of TTI is much more complex. Most increases in TTI are caused in large part by the pursuit of improved care. Imaging studies to better identify areas of tumor spread pre-treatment, transitions in care to allow treatment by a more experienced provider, additional scheduling to allow concurrent chemotherapy – all of these increase TTI but also improve patient outcomes. For this reason critical appraisal of the TTI literature is somewhat difficult. Some analyses do not report a significant survival detriment to TTI [24, 25] while others report significant detriments to rather short intervals [22, 26] (Table 1). By far the largest analysis of this issue demonstrated a significant detriment to delays longer than 60 days that further increased for the small subset of patients with TTI ≥ 91 days (HR 1.23, 1.15–1.32, *p* < 0.001) [27]. Evaluation of TTI as a continuous variable identified 67 days as the most critical threshold, with a TTI threshold of 46–52 days also appeared on 96% of the repeat simulations designed to test for validity. While these benchmarks are longer than the median TTI reported in a majority of the published literature, in 2011 in the Unites States 9.6% of patients had a TTI greater than 67 days and 25% of patients had a TTI of greater than 46 days with associated decreases in median overall survival from 71.9 months (TTI < 46–52 days) to 61 months (TTI 53–67 days) to 46.6 months (TTI > 67 days, *p* < 0.001) [27].

TTI is increasing in the United States and a prime reason for this increase is the pursuit of better care. This is not to say that treatment should begin in haste – patients need advanced imaging, pre-treatment dental attention, and increasingly complex treatment planning that takes time. However these necessary aspects of patient care can be completed without dangerously extended TTI. This need to coordinate multiple aspects of care in a limited amount of time is likely best accomplished by centers with high head and neck treatment volumes and likely in part responsible for said centers improved survival [28].

### Growth rate

While it is evident that cancer progresses over time, granular detail about growth rate is difficult to obtain. Thus, clinicians rarely directly observe this feature of tumor biology that is intricately linked to TTI. However, in an era of competition among different tumor types for limited resources (radiation treatment machine time or operating room time) the practice of definitive treatment of head and neck cancer with primary radiotherapy offers a simple mechanism to observe growth rate; most patients undergo an initial cross sectional imaging scan at the time of diagnosis and then have a similar scan performed for radiation treatment planning. Given the current state of TTI, these two scans are generally separated by a sufficiently long enough interval to allow for a measurement of tumor growth (Table 2).

**Table 2:** Growth rate analyses between diagnostic imaging scan and radiation treatment planning scan

	Institution	Era	N	Patients	Median scan interval	Growth rate	Survival
Waaijer [31]	UMC Utrecht	1996–2001	13	OPC	37 days	TVD: 96 days	
						Range 21–256	
Jensen [30]	Aarhus	2000–2005	61	Pharynx/Larynx/Oral Cavity	28 days	TVD: 99 days	
						Range 15 to > 234	
van Bockel [32]	UMC Utrecht	1996–2009	131	Larynx	26 days	HR for growth rate above median: 2.35	HR 1.93 (1.15–3.24) Dichotomous: −0.3ln (cc/day)
Murphy [29]	FCCC	2007–2014	85	OPC	35 days	TVD: 94 days	HR 1.94 (1.25–3.00)
						TSGR: 0.74%/day	Continuous
Perni [33]	MDACC	2004–2008	101	OPC	27 days	TVD: 112 days	HR 17.2 (5–75)
						TGV: 0.65%/day	Dichotomous: 1%/day

*OPC* = oropharynx cancer, *TVD* = tumor volume doubling time, *TSGR* = tumor specific growth rate, *TGV* = volumetric tumor growth velocity. TSGR and TGV are functionally the same

*In 2 analyses the impact of survival is not available. In the 3 analyses for which survival is reported this is from a multivariate analysis controlling for other risk factors potentially associated with survival

While predictors of a fast growth rate are not clear among different analyses, one oropharynx cancer dataset demonstrated that human papillomavirus (HPV)-negative disease grew at a faster rate than HPV-positive disease, with a suggestion that HPV-positive disease with a smoking history may also increase growth rate [29]. Other available datasets do not have enough HPV information to address this issue. Nonetheless, it is clear that the variation in growth rate among different subsites and even similar subsite head and neck tumors is extreme [29–33]. It is incumbent upon providers at all ends of the spectrum (primary care to tertiary care oncologist) to better appreciate which tumors in the clinic are expected to grow fast (and merit the most expedited oncologically safe treatment) and which are expected to plot a more middling pace. Although predictors of growth rate are not consistent, available estimates of tumor volume doubling time are somewhat consistent across different disease sites and treatment centers. Similarly, overall survival effects of delays noted are consistent – increased tumor growth rate is independently associated with worsening survival when controlling for other known risk factors. When one considers that a tumor may double in volume in roughly 90–100 days, the detriment of TTI exceeding 60 days becomes clearer.

### Treatment package time

Treatment package time refers to patients managed with primary surgery and adjuvant (chemo)radiation. The treatment package is the interval from the extirpative operation and the eventual completion of adjuvant therapy. Initial interrogation of this phenomenon suggested that patients whose radiation was initiated more than 6 weeks after surgery had poorer control rates than those whose radiation was begun earlier [34–36]. Other single institution reports cited radiation start within 7 weeks of an operation was beneficial, particularly for those patients with poor prognostic features [37]. This work is in part the reason that it is common for prospective adjuvant therapy investigations to mandate the initiation of (chemo)radiation within 4–8 weeks of an operation although it must be noted that many patients treated during the initial evaluation of time factors were treated with daily fractions of 1.8 Gy. A standard adjuvant course of 1.8 Gy daily radiation takes one week (7 days) longer than a standard course delivered in 2 Gy daily fractions (63 Gy in 1.8 Gy/fraction – 35 fractions v 60 Gy in 2.0 Gy/fraction - 30 fractions).

Given the suspicion that time factors most significantly influence the prognosis of high risk patients, a prospective study was conducted that evaluated accelerated adjuvant radiation alone in the setting of high risk resected head and neck cancer – both arms of the randomization received an equivalent adjuvant dose of 63 Gy, however it was administered in either 5 weeks via a concomitant boost technique or 7 weeks via daily fractionation. High risk patients treated in 5 weeks demonstrated a non-significant trend towards improved locoregional control (LRC) and overall survival (OS). Furthermore, the 5 week course was more forgiving with respect to the time interval between surgery and radiation. A prolonged interval in the time between surgery and radiation was significantly associated with worse LRC (*p* = 0.03) and OS (*p* = 0.01) for the patients treated over 7 weeks, while not significant for those treated according to the accelerated schedule. As a consequence, the package time was significant for both LRC and OS [38]. Unfortunately with increasing follow-up no difference in any outcome was appreciated in high-risk patients based on radiation fractionation schedule [39]. Given that the update failed to demonstrate a durable benefit to accelerated adjuvant therapy and the burgeoning application of adjuvant chemoradiation [40, 41] enthusiasm for the use of accelerated adjuvant therapy waned. The current standard of care in the adjuvant high risk setting is conventionally fractionated chemoradiation – the method to influence treatment package time is to maximize post-operative care and function so that patients are prepared to start adjuvant therapy expeditiously.

Similar to historic series of adjuvant radiation alone, a more modern population based analysis (> 50% of patients received adjuvant chemoradiation) continued to demonstrate the significant influence of treatment package time. In this analysis a significant improvement in overall survival was appreciated for those patients whose treatment was initiated within 42 days (6 weeks) of surgery when compared to those whose treatment did not begin for ≥50 days (HR 1.07; 95% CI, 1.02–1.12) [42]. Interestingly, given the lack of survival benefit appreciated in the definitive setting for accelerated chemoradiation [43], this analysis did demonstrate a survival benefit to modest acceleration.

### Radiation treatment time (RTT)

Most treatment prolongation is unplanned and likely due to acute toxicity – thus the results of prolonged RTT are difficult to study. By contrast the effects of treatment acceleration have been frequently investigated. The Radiation Therapy Oncology Group (RTOG) ran multiple trials investigating accelerated fractionation schedules for the definitive management of stage III/IV head and neck cancer with radiation alone, culminating in a large 4-arm trial of radiation alone (RTOG 9003) demonstrating, in part, that delivering 72 Gy in 6 weeks rather than 70 Gy in 7 weeks improved 2 year local-regional control by 8% [44]. Although proving that treatment in 6 weeks is superior to 7 is not the same as proving that treatment in 7 weeks is superior to 8, a logical conclusion was that an 8 week treatment of head and neck cancer was no longer considered acceptable [45]. Although RTOG 9003 demonstrated no OS improvement with acceleration, a subsequent large meta-analysis did demonstrate a survival benefit for patients receiving altered fractionation and this effect did not differ significantly according to tumor stage or tumor site [46]. Subsequent population based analyses similarly demonstrated a survival benefit to acceleration [47]. Thus the role of RTT in the setting of radiation alone is clear – modest acceleration of radiation results in a small but statistically significant improvement in LRC and OS.

The role of RTT in the setting of chemoradiation is less clear. Randomized trials suggest that the addition of concurrent chemotherapy improves the results of hyperfractionated definitive radiotherapy but acceleration of radiation does not enhance definitive concurrent chemoradiation [43, 48]. Given the lack of survival benefit to acceleration in the setting of concurrent chemotherapy and that accelerated radiation with concurrent chemotherapy has proven to be the most toxic mechanism of delivering non-surgical therapy [5], this treatment technique is not generally recommended. In 2018 acceleration is recommended in the setting of radiation alone, but not recommended in the setting of chemoradiation.

Prolonged RTT was evaluated in a population based analysis. On the contrary to treatment acceleration, this demonstrated that prolongation (defined as a definitive radiation course taking ≥56 days to completion) resulted in worsening survival in both the radiation alone (HR 1.45 95% CI 1.16–1.83 *p* = 0.0013) and chemoradiation settings (HR 1.22 95% CI 1.01–1.46 *p* = 0.0368). Prolonged RTT has many causes, but this series was limited to patients who completed therapy. Thus, patients who expired during treatment or did not finish their radiation course as prescribed are not included. In view of this study’s generous definition of prolongation (treatment needed to be extended at least a week prior to be classified as ‘prolonged’) it would require unsustainable acceleration to mitigate the effect of a prolonged RTT (increased dose per fraction and/or increased fractions in a given week [49]. The best way to address prolonged RTT is through prevention.

## Conclusion

The management of head and neck cancer is complex. Many tumors present locally advanced, and require a team of professionals to adequately diagnose, stage, and treat the diagnosis. Even in the most favorable setting completing the necessary pre-treatment tasks expeditiously is difficult – unfortunately given the rarity of the disease many patients must travel at least 50 miles to be evaluated by their oncology provider. In addition the treatment frequently requires fractionated radiation with high acute morbidity making daily presentation to the cancer center difficult. All of these factors (and others) contribute to make the timely completion of a treatment package difficult.

Logic would suggest that the aforementioned difficulty in the expeditious initiation and completion of therapy could be ameliorated by familiarity; institutions that treat a high volume of head and neck cancer are likely better at the delivery of swift, high quality care. This would be expected to improve outcomes. Recent large data analyses support this premise. Patients treated on RTOG 0129 at historically low-accruing head and neck centers (median accrual to 21 trials from 1997 to 2002: 4) had significantly worse 5-year survival (51% v 69%, *P* = 0.002) than those treated at historically high accruing centers (median accrual on same trials: 65) despite having somewhat more favorable stage and performance status [28]. In addition, this analysis demonstrated that patients treated at historically low-accruing centers had a significantly longer RTT. A subsequent big data analysis defining a high-volume facility as the top 1% of centers by the number of patients treated similarly demonstrated an improvement in 5-year overall survival for high volume facilities (61.6% v 55.5%, *P* < 0.001) although timing was not specifically evaluated [50]. Finally, other analyses evaluating radiation quality on a randomized trial [51] and intensity modulated RT volume by provider [52] have suggested benefits to treatment at high volume head and neck cancer centers. Although timing is not evaluated in these analyses, it stands to reason that a component of the benefit seen is secondary to better appreciation of the importance of timing at these centers.

As this monograph demonstrates – however difficult it may be treatment timing is important. It takes dedication and attention from a committed multidisciplinary team to treat head and neck cancer efficiently and correctly. Currently the most detailed data of this phenomenon identifies 46 days from histologic diagnosis to treatment initiation as a benchmark. While this seems like a generous amount of time, care transitions often result in a 20–30 day interval between histologic diagnosis and first meeting with the provider (surgeon/radiation oncologist/medical oncologist) who will actually deliver care. Providers must not only perform the correct tasks in the correct order – they also must be done in parallel (i.e. visit to the dentist and staging imaging scan on the same day). Expediting treatment start and completion represents a simple, cost-effective mechanism to improve patient outcomes.

## Abbreviations

CI: Confidence interval
CT: Computed tomography
FDG/PET: Fluorodeoxyglucose/positron emission tomography
HNSCC: Head and neck squamous cell carcinoma
HPV: Human papillomavirus
HR: Hazard ratio
IMRT: Intensity modulated radiation therapy
LRC: Locoregional control
OS: Overall survival
RTOG: Radiation Therapy Oncology Group
RTT: Radiation treatment time
TTI: Time to treatment initiation
